# Severe Multifactorial Lactic Acidosis With Pulmonary Edema and Myocardial Injury in an Elderly Patient on Metformin Managed Conservatively: A Case Report

**DOI:** 10.7759/cureus.102890

**Published:** 2026-02-03

**Authors:** Kyaw Zin Aung, Ei Ei Cho, Su Su Htun, Nay Aung Zin, Thinn Thiri Soe, Han Thant Thant, Cherry Myint, Naw Eh Law Saw

**Affiliations:** 1 Internal Medicine, Kulhudhuffushi Regional Hospital, Kulhudhuffushi City, MDV; 2 Orthopaedics and Traumatology, Kulhudhuffushi Regional Hospital, Kulhudhuffushi City, MDV; 3 Emergency, Kulhudhuffushi Regional Hospital, Kulhudhuffushi City, MDV

**Keywords:** acute pulmonary edema, chronic kidney disease, conservative management (without dialysis), metformin-associated lactic acidosis, sepsis–induced lactic acidosis, type 2 myocardial infarction

## Abstract

Severe lactic acidosis is a life-threatening condition in elderly patients with multiple comorbidities. We report a 90-year-old man with type 2 diabetes mellitus (HbA1c 7.1%), hypertension, and chronic kidney disease (CKD) stage 3a (baseline estimated glomerular filtration rate (eGFR) of 56 mL/min/1.73 m²) who presented with acute dyspnea, orthopnea, and oliguria. Laboratory evaluation revealed metabolic acidaemia (pH 7.22), marked hyperlactatemia (8 mmol/L), and acute-on-chronic kidney injury. Clinical findings were consistent with acute pulmonary edema and myocardial injury, with elevated cardiac biomarkers but no ischemic electrocardiographic changes. Lactic acidosis was multifactorial, driven by sepsis from a diabetic foot infection, renal dysfunction, and metformin as a potential contributor. Initial management included intravenous sodium bicarbonate, loop diuretics, ventilatory support, and antimicrobial therapy. Renal replacement therapy was declined by the patient, and intensive conservative management with close biochemical and clinical monitoring was pursued. The patient gradually improved, with normalization of acid-base status, renal function, lactate, and cardiac biomarkers, allowing successful extubation and transition to oral therapy. This case highlights the diagnostic and therapeutic challenges of severe lactic acidosis in elderly patients with multiple comorbidities and demonstrates that individualized supportive care can lead to favorable outcomes even when standard interventions such as dialysis are not feasible.

## Introduction

Lactic acidosis is a serious and potentially life-threatening metabolic disturbance characterized by elevated serum lactate levels and metabolic acidemia. It commonly arises in the context of tissue hypoperfusion, sepsis, acute organ dysfunction, or impaired lactate clearance and is associated with increased morbidity and mortality, particularly in elderly and critically ill patients [1,2]. Sepsis is a frequent precipitant, causing lactic acidosis through mechanisms that extend beyond simple hypoperfusion, including mitochondrial dysfunction, inflammatory metabolic reprogramming, and impaired lactate clearance, even in the absence of overt hypotension [2].

In patients with type 2 diabetes mellitus, lactic acidosis may occasionally occur in those receiving metformin, particularly when additional risk factors such as advanced age, acute illness, or renal dysfunction are present [3,4]. However, true metformin-associated lactic acidosis is rare, difficult to confirm in routine clinical practice, and frequently confounded by concurrent conditions that independently predispose to lactate accumulation [3,5]. In this context, attributing lactic acidosis solely to metformin without objective drug measurements can be misleading [4,6].

We report a case of severe multifactorial lactic acidosis in a 90-year-old man with type 2 diabetes mellitus, chronic kidney disease (CKD), and diabetic foot sepsis. While metformin-associated lactic acidosis was considered as a differential, it could not be established. This case highlights the diagnostic complexity and therapeutic challenges of managing severe lactic acidosis in very elderly patients and underscores the importance of individualized, patient-centered supportive care.

## Case presentation

A 90-year-old man with a 16-year history of type 2 diabetes mellitus, hypertension, and a three-year history of CKD (baseline estimated glomerular filtration rate (eGFR) 56 mL/min/1.73 m², CKD stage 3a) presented to the emergency department with acute-onset dyspnea, orthopnea, and oliguria of one day’s duration. He denied fever, cough, chest pain, or antecedent exertional dyspnea. He had been taking metformin 500 mg twice daily since his diabetes diagnosis, with good adherence; the last dose was taken approximately eight hours before admission. His HbA1c at admission was 7.1%, reflecting controlled diabetes. There was no history of overdose or recent dose escalation. He denied recent use of nephrotoxic or lactate-promoting agents, including nonsteroidal anti-inflammatory drugs, iodinated contrast, alcohol, or sodium-glucose cotransporter 2 inhibitors, and there had been no recent changes in angiotensin-converting enzyme inhibitors or angiotensin receptor blockers.

On presentation, he was in moderate respiratory distress with tachypnea (38/min), hypoxemia (SpO₂ 84% on room air), and tachycardia (HR 110/min). Elevated jugular venous pressure, bilateral basal crepitations, and a gallop rhythm were noted. Neurological examination was unremarkable (GCS 15/15).

Laboratory evaluation revealed severe metabolic acidaemia (arterial pH 7.22, bicarbonate 12 mmol/L) with marked hyperlactatemia (8 mmol/L) and acute-on-chronic kidney injury (serum creatinine 1.8 mg/dL). The calculated anion gap was 22 mmol/L. Urinary and serum ketones were negative, excluding diabetic ketoacidosis. Random blood glucose was 221 mg/dL.

Sepsis evaluation revealed a mildly elevated procalcitonin level (1.1 ng/mL). Blood cultures were obtained and remained negative. A diabetic foot infection with local inflammatory changes was identified as the likely source of sepsis, and subsequent wound swab culture grew Staphylococcus aureus.

**Figure 1:**
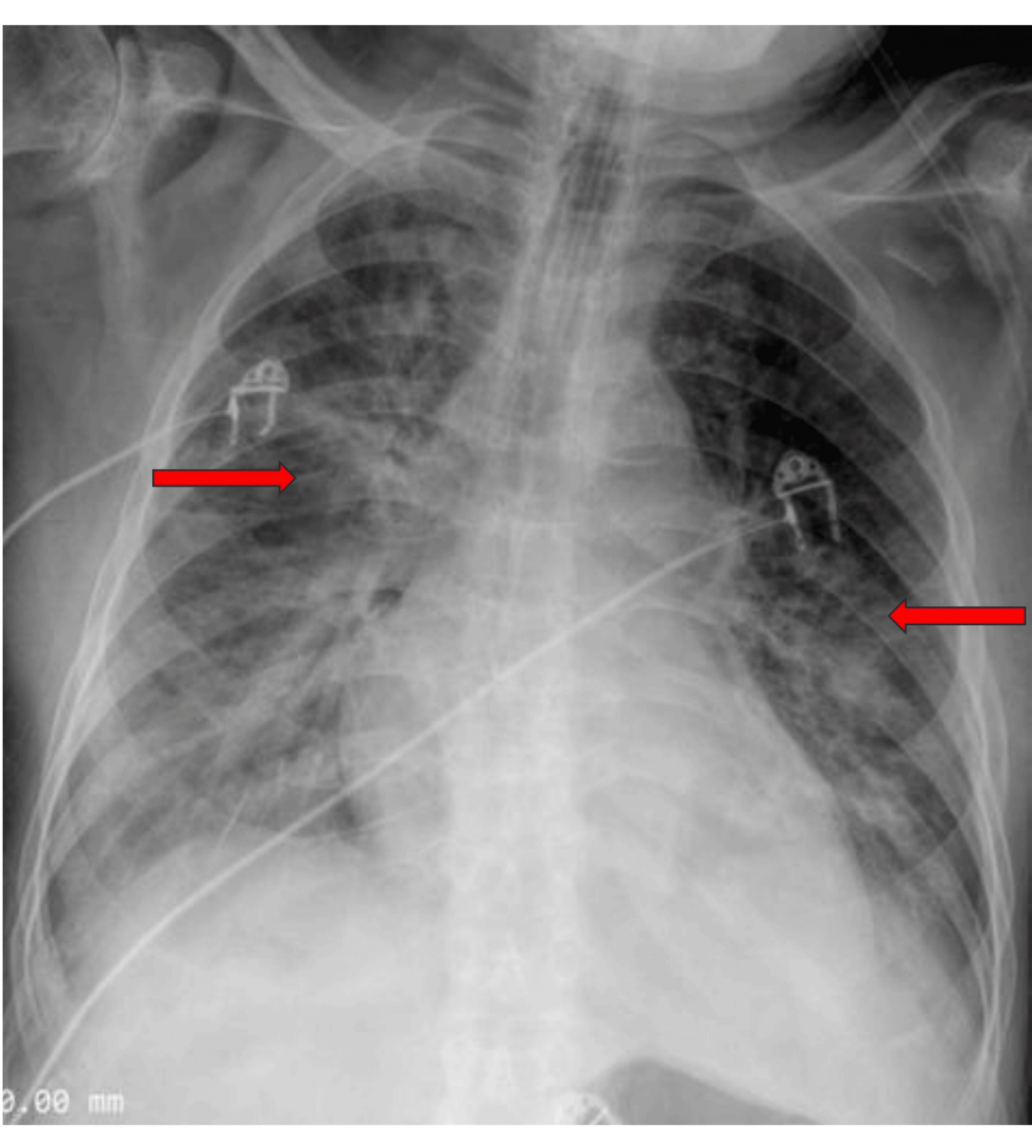
Portable anteroposterior chest radiograph showing bilateral perihilar alveolar opacities in a bat-wing distribution, consistent with acute pulmonary edema.

**Figure 2:**
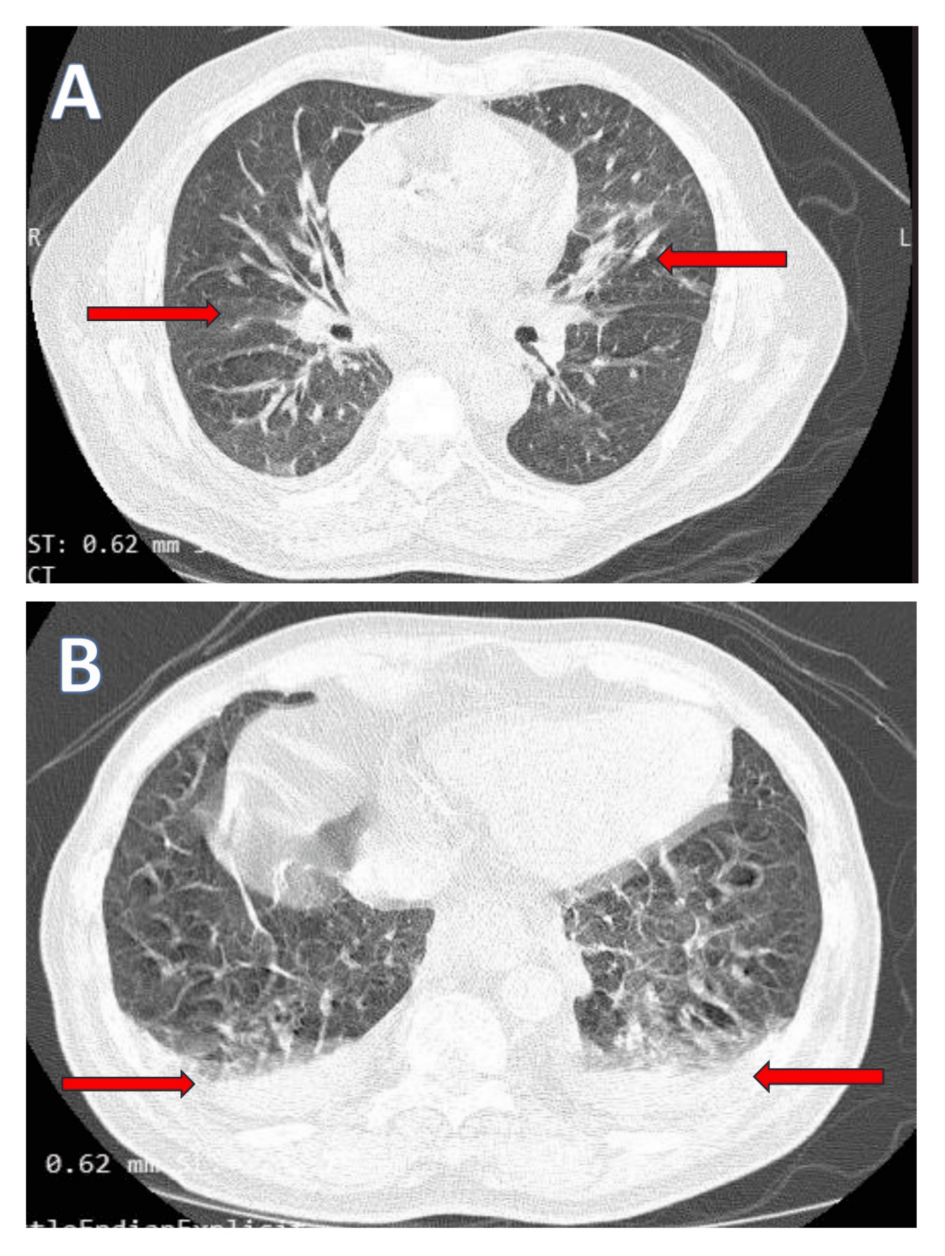
High-resolution computed tomography (HRCT) of the chest demonstrating features of acute cardiogenic pulmonary edema. (A) Axial image at the level of the hila shows bilateral symmetric perihilar ground-glass opacities with interlobular septal thickening (arrows), forming a bat-wing pattern. (B) Axial image at the lung bases demonstrates bilateral small dependent pleural effusions (arrows) with adjacent posterior basal ground-glass opacities.

Electrocardiography demonstrated sinus tachycardia without ischemic changes. Chest radiography and high-resolution computed tomography revealed bilateral perihilar alveolar opacities and interlobular septal thickening, consistent with acute cardiogenic pulmonary edema (Figures 1, 2). Transthoracic echocardiography showed a normal-sized left ventricle with preserved systolic function, mild global hypokinesia, grade I diastolic dysfunction, and borderline left atrial enlargement. No regional wall motion abnormalities or pericardial effusion were observed.

Metformin and vildagliptin were discontinued on admission. Initial management included intravenous sodium bicarbonate (50 mL of 7% solution diluted in 50 mL normal saline over 30 minutes), intravenous furosemide (80 mg bolus followed by continuous infusion at 5 mg/hour), glycerin trinitrate infusion, and supplemental oxygen therapy. Despite partial early improvement, the patient developed worsening metabolic derangements, with serum lactate rising to 13.1 mmol/L and arterial pH declining to 7.145. Due to refusal of renal replacement therapy, repeated intravenous sodium bicarbonate (20 mL of 7% solution diluted in 30 mL normal saline every six hours) was administered guided by serial arterial blood gas analyses. Progressive respiratory failure necessitated endotracheal intubation and mechanical ventilation.

Empirical antimicrobial therapy with intravenous ceftriaxone and oral clindamycin (300 mg four times daily) was initiated for diabetic foot sepsis, and antibiotic therapy was adjusted according to sensitivity results. Glycemic control during hospitalization was achieved using a combination of variable-rate intravenous insulin infusion and then basal-bolus subcutaneous insulin, targeting blood glucose levels between 140 and 180 mg/dL.

Over the ensuing days, the patient demonstrated gradual improvement in acid-base balance, serum lactate, renal function, urine output, and cardiac biomarkers (Table 1). Blood glucose remained within the target range. He was successfully extubated, transitioned from continuous to intermittent intravenous diuretics, and subsequently maintained on oral therapy. The patient remained clinically stable at discharge with improving laboratory parameters.

**Table 1:** Summary of laboratory investigations at admission and on subsequent follow-up days CRP: C-reactive protein; CK: creatine kinase; CK MB: creatine kinase – myocardial band; LDH:  lactate dehydrogenase; Cr: creatinine; BUN: blood urea nitrogen; HCO3: bicarbonate; PaO₂: partial pressure of oxygen; PaCO₂: partial pressure of carbon dioxide. Day 1 refers to the day of admission; subsequent days follow accordingly. Day 1 includes morning and evening values due to rapid clinical deterioration.

Parameters	Day 1		Day 2	Day 3	Day 4	Day 5	Day 6	Day 7	Day 8	Day 9	Reference
	Morning	Evening									
CRP (mg/L)	44	40.8	35	31.1	50.3	88.2	164	68.4	22.1	12.6	<10
Troponin I (ng/mL)	0.012	0.02	0.039	0.33	8.9	16.88	8.17	5.89	5.78	3.47	<0.02
CK (U/L)	136	124	117	3240	5188	12718	9965	5241	1145	628	55-170
CK MB (U/L)	14	12	12	44	65	72	50	31	22	15	0-16
LDH (U/L)	142	136	201	298	591	912	751	612	468	376	120-246
Cr (mg/dL)	1.8	1.7	1.6	2.4	2.9	2.5	2.2	1.9	1.6	1.8	0.52-1.04
BUN (mg/dL)	25.2	24.2	26.2	25.2	33.6	37.9	36.9	38.8	32.2	33.2	7.0-17.0
Serum lactate (mmol/L)	8	13.1	9.8	2.37	2	1.85	1.81	1.5	1.2	0.8	0.5-2
Serum HCO3 (mmol/L)	12	12.3	17.4	27.1	27.6	29.1	24.4	22.5	26.4	26	22-28
pH	7.22	7.145	7.306	7.43	7.406	7.449	7.422	7.44	7.45	7.42	7.35-7.45
PaO_2_ (mmHg)	60	68	69	122	173	80	84	99	102	102	83-108
PaCO_2_ (mmHg)	32.7	33.9	33.4	40.8	28.8	31.9	41.9	32.4	34.8	38.2	32-48

## Discussion

Severe lactic acidosis in elderly patients is frequently multifactorial, reflecting the combined effects of chronic comorbidities, acute systemic insults, and age-related reductions in physiological reserve. In the present case, advanced age, sepsis secondary to a diabetic foot infection, acute-on-chronic kidney injury, and probable systemic hypoperfusion were the most plausible drivers of lactic acidosis. Although the patient was receiving metformin, metformin-associated lactic acidosis could not be definitively established. Serum metformin levels were unavailable, and given the therapeutic dosing, preserved baseline renal function, and the presence of clear alternative precipitants, MALA was unlikely to represent the primary etiology [3,4,6].

Sepsis is a well-recognized cause of lactic acidosis and can elevate lactate levels through mechanisms beyond tissue hypoxia, including mitochondrial dysfunction, inflammatory metabolic reprogramming, and impaired lactate clearance [2,5]. In this patient, progressive hyperlactatemia and severe acidemia occurred alongside evidence of infection and organ dysfunction, supporting sepsis-related lactic acidosis as the dominant process. Severe acidemia itself can further exacerbate cardiovascular dysfunction by depressing myocardial contractility, reducing responsiveness to catecholamines, and increasing the risk of arrhythmias [1,2]. These pathophysiologic effects likely contributed to the transient myocardial injury and acute pulmonary edema observed, in the absence of ischemic electrocardiographic changes.

Renal replacement therapy is often recommended in cases of severe lactic acidosis with refractory acidemia or significant renal impairment, as it facilitates lactate and acid removal and corrects metabolic derangements [1,6]. In this case, however, renal replacement therapy was declined after informed discussion, reflecting the patient’s preferences and goals of care. Intensive supportive management, including repeated bicarbonate therapy guided by serial arterial blood gas analysis, careful diuretic use, ventilatory support, and prompt antimicrobial therapy, resulted in gradual correction of acid-base abnormalities, resolution of hyperlactatemia, and recovery of renal function. Similar favorable outcomes have been reported in selected patients managed without dialysis when close monitoring and aggressive treatment of underlying causes are ensured [6].

This case highlights several key clinical lessons. First, lactic acidosis in elderly patients with diabetes should be approached as a multifactorial condition rather than prematurely attributed to metformin [3,4]. Second, diagnostic uncertainty is common when serum metformin levels are unavailable, underscoring the need for comprehensive evaluation of infectious, renal, and cardiovascular contributors [6]. Finally, individualized supportive care that integrates patient preferences, multidisciplinary decision-making, and vigilant monitoring can achieve favorable outcomes even when standard interventions such as renal replacement therapy are not pursued.

## Conclusions

Severe lactic acidosis in elderly patients often results from multiple factors, including comorbidities, acute illness, and medications such as metformin. Prompt recognition and careful management of all contributing factors are essential. Although renal replacement therapy is generally recommended in severe cases, intensive supportive care with correction of acid base disturbances, careful fluid and ventilatory management, and treatment of underlying infections can achieve favorable outcomes when dialysis is declined or not feasible. This case demonstrates that individualized, multidisciplinary care that respects patient preferences and clinical context can lead to recovery even in critically ill elderly patients with multifactorial lactic acidosis.

## References

[1] Kraut JA, Madias NE (2014). Lactic acidosis. N Engl J Med.

[2] Rodríguez-Villar S, Kraut JA, Arévalo-Serrano J (2021). Systemic acidemia impairs cardiac function in critically Ill patients. EClinicalMedicine.

[3] Salpeter SR, Greyber E, Pasternak GA, Salpeter EE (2003). Risk of fatal and nonfatal lactic acidosis with metformin use in type 2 diabetes mellitus: systematic review and meta-analysis. Arch Intern Med.

[4] Lalau JD, Arnouts P, Sharif A, De Broe ME (2015). Metformin and other antidiabetic agents in renal failure patients. Kidney Int.

[5] Mizock BA, Falk JL (1992). Lactic acidosis in critical illness. Crit Care Med.

[6] Seidowsky A, Nseir S, Houdret N, Fourrier F (2009). Metformin-associated lactic acidosis: a prognostic and therapeutic study. Crit Care Med.

